# The genome sequence of the Brown Silverhorn, *Athripsodes cinereus *(Curtis, 1834)

**DOI:** 10.12688/wellcomeopenres.19765.1

**Published:** 2023-09-01

**Authors:** Ian Wallace, John DS Findlay, Benjamin W. Price, Natural History Museum Genome Acquisition Lab, Darwin Tree of Life Barcoding collective, Wellcome Sanger Institute Tree of Life programme, Wellcome Sanger Institute Scientific Operations: DNA Pipelines collective, Tree of Life Core Informatics collective, Darwin Tree of Life Consortium

**Affiliations:** 1 British Caddis Recording Scheme, Wirral, England, UK; 2 Environment Agency, Huntingdon, England, UK; 3 Natural History Museum, London, England, UK

**Keywords:** Athripsodes cinereus, the Brown Silverhorn, genome sequence, chromosomal, Trichoptera

## Abstract

We present a genome assembly from an individual male *Athripsodes cinereus* (the Brown Silverhorn; Arthropoda; Insecta; Trichoptera; Leptoceridae). The genome sequence is 716.2 megabases in span. Most of the assembly is scaffolded into 25 chromosomal pseudomolecules, including the Z sex chromosome. The mitochondrial genome has also been assembled and is 14.93 kilobases in length. Gene annotation of this assembly on Ensembl identified 16,582 protein coding genes.

## Species taxonomy

Eukaryota; Metazoa; Eumetazoa; Bilateria; Protostomia; Ecdysozoa; Panarthropoda; Arthropoda; Mandibulata; Pancrustacea; Hexapoda; Insecta; Dicondylia; Pterygota; Neoptera; Endopterygota; Amphiesmenoptera; Trichoptera; Integripalpia; Brevitentoria; Leptoceroidea; Leptoceridae; Leptocerinae; Athripsodini; Athripsodes (Curtis, 1834) (NCBI:txid446526).

## Background

*Athripsodes cinereus*, the Brown Silverhorn, is a common caddis throughout Britain ( NBN Trust, 2023). This species is in the family Leptoceridae, which are characterised by having very long antennae. The larvae of *A. cinereus* live in permanent stony rivers, large streams and on lake shores, and can occasionally be found on coarse sandy bottoms of large ponds and canals. They are mainly littoral but are regularly taken where the water depth is up to 10 metres. Pupae, emerging from cases on the bottom, swim to the water surface where the adult emerges. Larvae feed by grazing algae and other items from surfaces, and the gut analysis of larger larvae reveals that items such as chironomid larvae are always a significant component ( Higler, 2005).

Full grown larvae are found from spring to early autumn and adults have been found from May to October. This is quite a small caddis, and it would seem possible for larvae hatching in late spring to mature in a few weeks, but there is no organised second generation and there is probably normally only one generation a year. In winter there is little growth of its grazed food and consequently little larval growth and most larvae are half-grown or less over that season.

Swarming and mating behaviour is the best-studied aspect of *A. cinereus* biology. The males join day-time swarms around features of the waterbody and its banks. Each male flies about one and a half metres, rapidly from side to side and a few centimetres above the water surface; the whole swarm is about five metres across. Females are attracted to the swarm by visual signs only and males are attracted to the arriving females. A male and female grab each other and at that moment the female stops flying and he must carry her upwards to bankside vegetation where they copulate for a few minutes ( Gullefors & Petersson, 1993). If the male runs out of energy and they land on the water surface, the female will attempt to escape, as the male has, in a real sense, failed a male fitness test ( Petersson, 1995). It is clearly advantageous for a male to be on the outside of the swarm, and they jostle and collide to stay there, and a male will only be there in prime condition for a couple of days.

Shortly after copulation the female is ready to lay. The unexpanded egg mass is extruded and held by flaps against a flat plate at the end of the abdomen. The insect flies over the water surface and will choose a spot in which to dip the tip of the abdomen, whilst in flight. This dislodges the egg ball that sinks, the jelly surrounding the eggs absorbs water and swells, and the mass becomes stuck to an object such as a stone or vegetation. After about two weeks the eggs hatch. The newly hatched larvae do not seem to have an organised planktonic stage before starting to make a case ( Hanna, 1961). The female will lay two or sometimes more egg masses, and mates afresh before each egg-laying event ( Petersson, 1990).

The adults are distinctive, but the markings are a little variable. Most adult records are made during the day, but it is also common at light. They can be identified in the field using the key in Wallace *et al.* (2022), but confirmation from genitalia examination of preserved specimens may be required for some individuals. Examination of head markings using a hand lens or microscope is needed to identify larvae, and this necessitates them being killed or anaesthetised. Wallace *et al*. (2003) provide a definitive key, but there is no key to identify eggs or pupae.

The genome of *Athripsodes cinereus* was sequenced as part of the Darwin Tree of Life Project, a collaborative effort to sequence all named eukaryotic species in the Atlantic Archipelago of Britain and Ireland. Here we present a chromosomally complete genome sequence for *Athripsodes cinereus*, based on one specimen from the River Kym at Great Staughton.

## Genome sequence report

**Figure 1.**
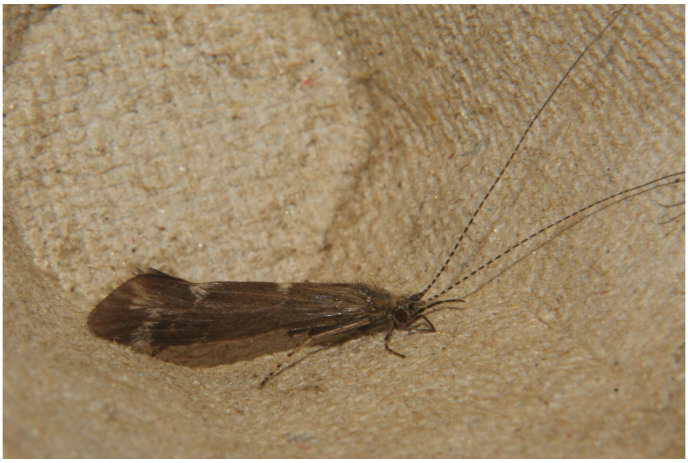
Photograph of an *Athripsodes cinereus* © Philip Mark Osso.

The genome was sequenced from one male *Athripsodes cinereus* ( Figure 1) collected from Great Staughton, UK (52.27, –0.35). A total of 36-fold coverage in Pacific Biosciences single-molecule HiFi long reads was generated. Primary assembly contigs were scaffolded with chromosome conformation Hi-C data. Manual assembly curation corrected 225 missing joins or mis-joins and removed 49 haplotypic duplications, reducing the assembly length by 0.71% and the scaffold number by 33.6%, and decreasing the scaffold N50 by 6.08%.

**Figure 2.**
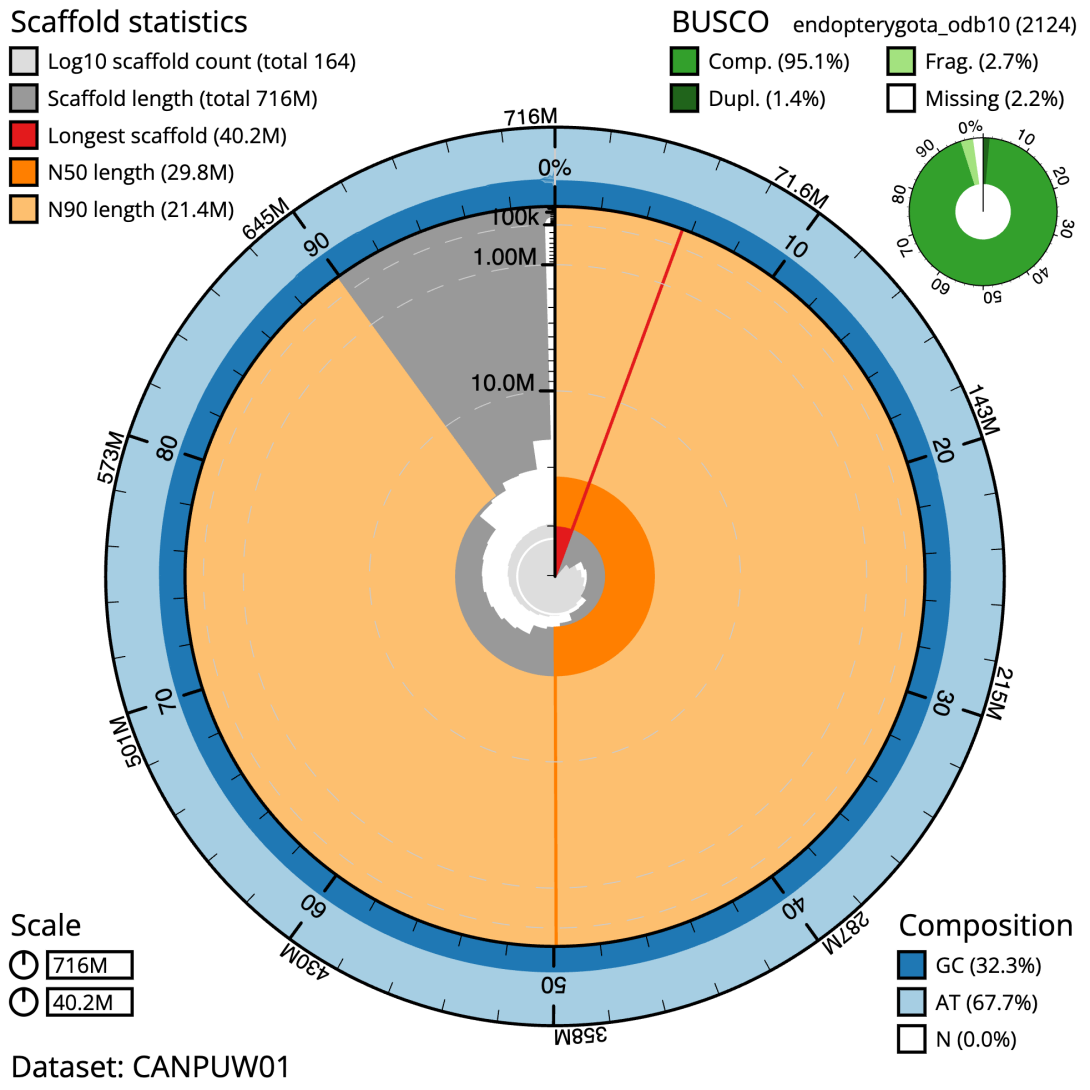
Genome assembly of *Athripsodes cinereus*, iiAthCine2.1: metrics. The BlobToolKit Snailplot shows N50 metrics and BUSCO gene completeness. The main plot is divided into 1,000 size-ordered bins around the circumference with each bin representing 0.1% of the 716,257,483 bp assembly. The distribution of scaffold lengths is shown in dark grey with the plot radius scaled to the longest scaffold present in the assembly (40,209,954 bp, shown in red). Orange and pale-orange arcs show the N50 and N90 scaffold lengths (29,754,657 and 21,428,489 bp), respectively. The pale grey spiral shows the cumulative scaffold count on a log scale with white scale lines showing successive orders of magnitude. The blue and pale-blue area around the outside of the plot shows the distribution of GC, AT and N percentages in the same bins as the inner plot. A summary of complete, fragmented, duplicated and missing BUSCO genes in the endopterygota_odb10 set is shown in the top right. An interactive version of this figure is available at https://blobtoolkit.genomehubs.org/view/iiAthCine2.1/dataset/CANPUW01/snail.

The final assembly has a total length of 716.2 Mb in 164 sequence scaffolds with a scaffold N50 of 29.8 Mb ( Table 1). Most (99.44%) of the assembly sequence was assigned to 25 chromosomal-level scaffolds, representing 24 autosomes and the Z sex chromosome. Chromosome-scale scaffolds confirmed by the Hi-C data are named in order of size ( Figure 2– Figure 5; Table 2). While not fully phased, the assembly deposited is of one haplotype. Contigs corresponding to the second haplotype have also been deposited. The mitochondrial genome was also assembled and can be found as a contig within the multifasta file of the genome submission.

**Table 1.** Genome data for *Athripsodes cinereus*, iiAthCine2.1.

Project accession data		
Assembly identifier	iiAthCine2.1	
Species	*Athripsodes cinereus*	
Specimen	iiAthCine2	
NCBI taxonomy ID	446526	
BioProject	PRJEB57670	
BioSample ID	SAMEA7521123	
Isolate information	iiAthCine2: whole organism (DNA sequencing) iiAthCine4: whole organism (Hi-C scaffolding)	
Assembly metrics *		*Benchmark*
Consensus quality (QV)	59.3	*≥ 50*
*k*-mer completeness	100%	*≥ 95%*
BUSCO **	C:95.1%[S:93.7%,D:1.4%], F:2.7%,M:2.2%,n:2,124	*C ≥ 95%*
Percentage of assembly mapped to chromosomes	99.44%	*≥ 95%*
Sex chromosomes	Z chromosome	*localised homologous pairs*
Organelles	Mitochondrial genome assembled	*complete single alleles*
Raw data accessions		
PacificBiosciences SEQUEL II	ERR10499357	
Hi-C Illumina	ERR10501023	
Genome assembly		
Assembly accession	GCA_947579605.1	
*Accession of alternate haplotype*	GCA_947579385.1	
Span (Mb)	716.2	
Number of contigs	1418	
Contig N50 length (Mb)	0.9	
Number of scaffolds	163	
Scaffold N50 length (Mb)	29.8	
Longest scaffold (Mb)	40.2	
Genome annotation		
Number of protein-coding genes	16,582	
Number of gene transcripts	16,603	

* Assembly metric benchmarks are adapted from column VGP-2020 of “Table 1: Proposed standards and metrics for defining genome assembly quality” from ( Rhie *et al.*, 2021). ** BUSCO scores based on the endopterygota_odb10 BUSCO set using v5.3.2. C = complete [S = single copy, D = duplicated], F = fragmented, M = missing, n = number of orthologues in comparison. A full set of BUSCO scores is available at https://blobtoolkit.genomehubs.org/view/iiAthCine2.1/dataset/CANPUW01/busco.

**Figure 3.**
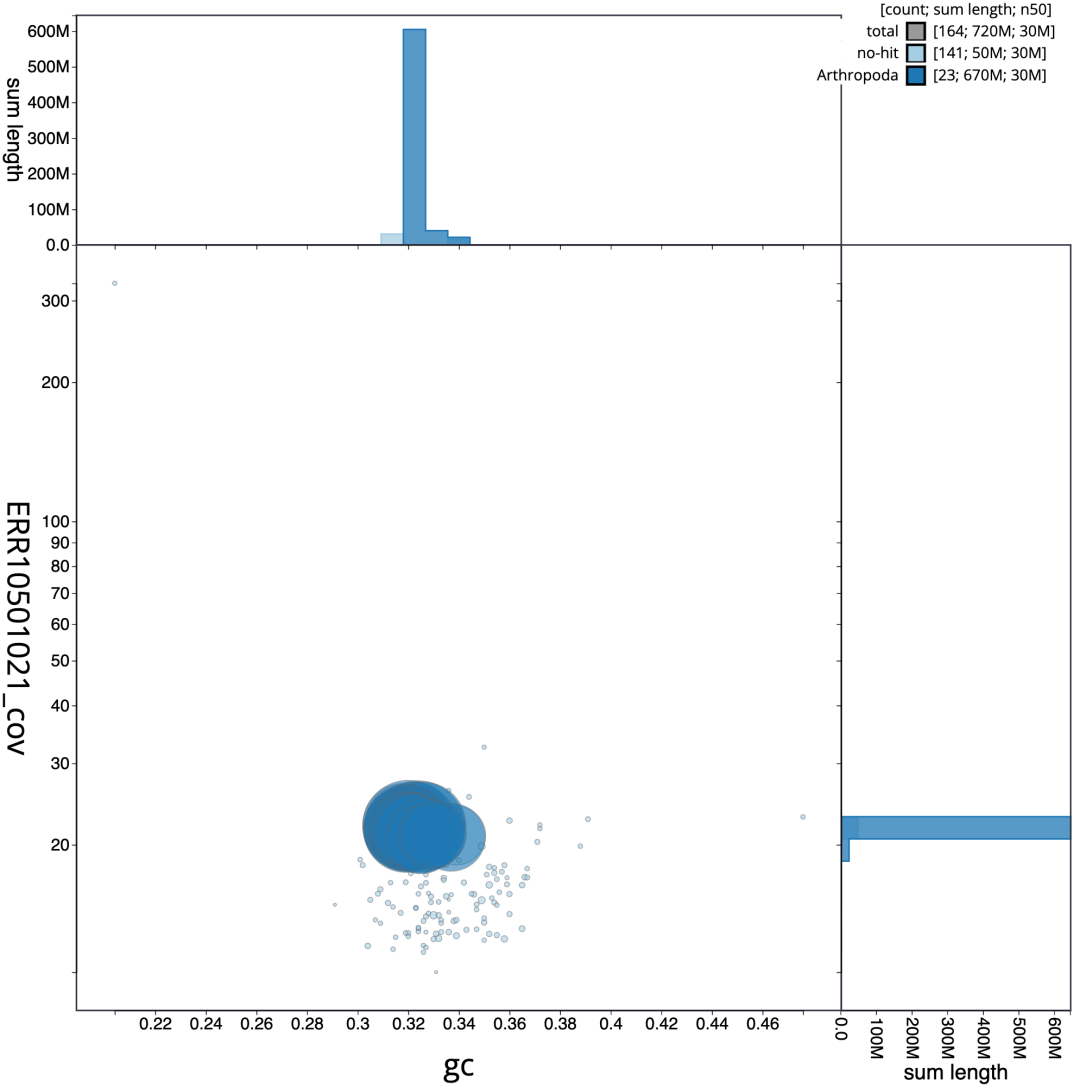
Genome assembly of *Athripsodes cinereus*, iiAthCine2.1: BlobToolKit GC-coverage plot. Scaffolds are coloured by phylum. Circles are sized in proportion to scaffold length. Histograms show the distribution of scaffold length sum along each axis. An interactive version of this figure is available at https://blobtoolkit.genomehubs.org/view/iiAthCine2.1/dataset/CANPUW01/blob.

**Figure 4.**
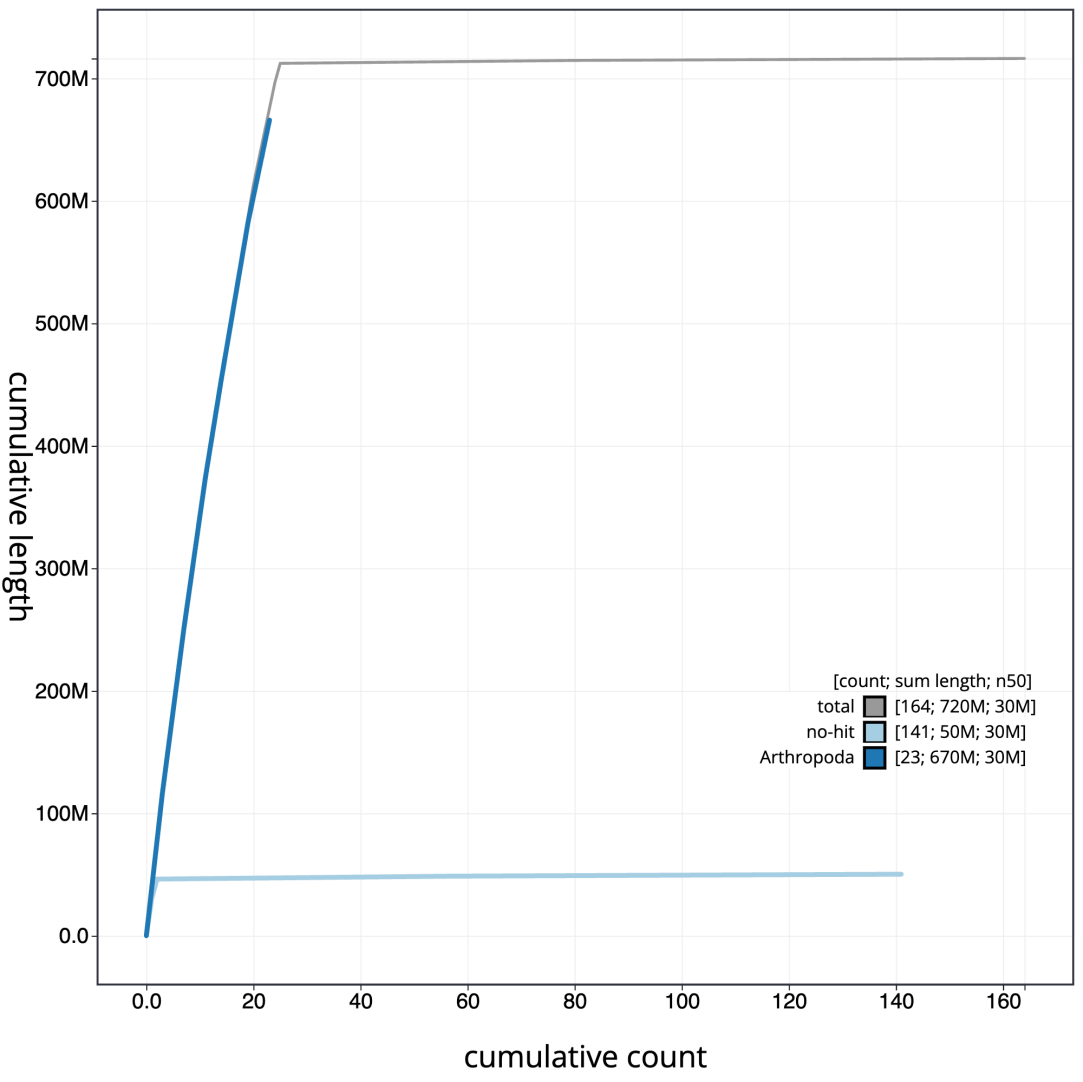
Genome assembly of *Athripsodes cinereus*, iiAthCine2.1: BlobToolKit cumulative sequence plot. The grey line shows cumulative length for all scaffolds. Coloured lines show cumulative lengths of scaffolds assigned to each phylum using the buscogenes taxrule. An interactive version of this figure is available at https://blobtoolkit.genomehubs.org/view/iiAthCine2.1/dataset/CANPUW01/cumulative.

**Figure 5.**
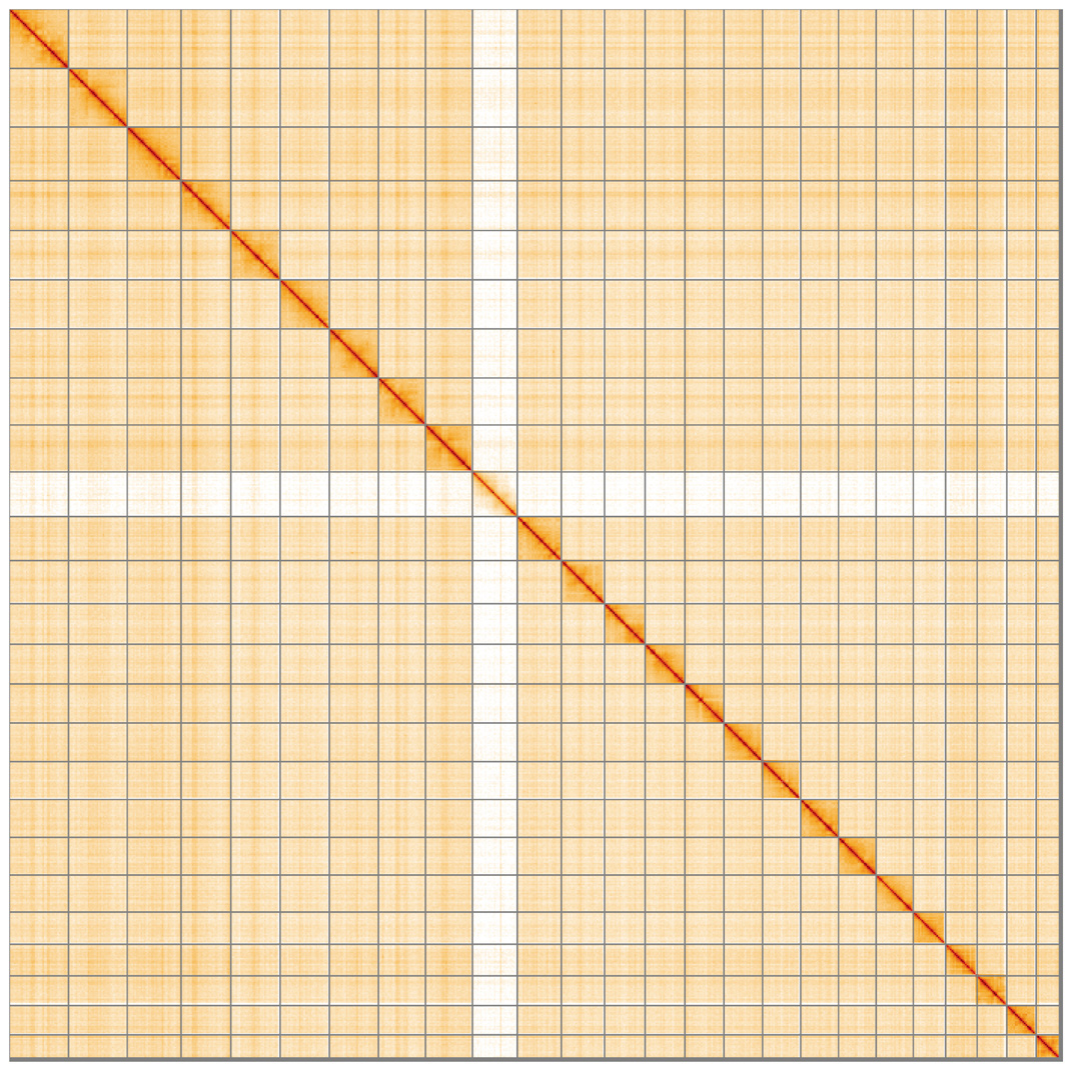
Genome assembly of *Athripsodes cinereus*, iiAthCine2.1: Hi-C contact map of the iiAthCine2.1 assembly, visualised using HiGlass. Chromosomes are shown in order of size from left to right and top to bottom. An interactive version of this figure may be viewed at https://genome-note-higlass.tol.sanger.ac.uk/l/?d=ZK1MgaY-ThSzMAiu5sZrbw.

**Table 2.** Chromosomal pseudomolecules in the genome assembly of *Athripsodes cinereus*, iiAthCine2.

INSDC accession	Chromosome	Length (Mb)	GC%
OX388305.1	1	40.21	32.5
OX388306.1	2	39.82	32.0
OX388307.1	3	36.54	32.0
OX388308.1	4	33.79	32.5
OX388309.1	5	33.38	32.0
OX388310.1	6	33.37	32.0
OX388311.1	7	33.31	32.0
OX388312.1	8	31.93	32.0
OX388313.1	9	31.83	32.0
OX388314.1	10	29.75	32.0
OX388315.1	11	29.3	32.0
OX388316.1	12	27.48	32.0
OX388317.1	13	27.05	32.0
OX388318.1	14	26.51	32.0
OX388319.1	15	26.14	32.0
OX388320.1	16	25.79	32.0
OX388321.1	17	25.69	32.0
OX388322.1	18	25.47	32.0
OX388323.1	19	25.29	32.5
OX388324.1	20	21.82	32.5
OX388325.1	21	21.43	33.5
OX388326.1	22	20.16	33.0
OX388327.1	23	19.94	33.0
OX388328.1	24	15.83	34.0
OX388329.1	Z	30.47	32.0
OX388330.1	MT	0.01	20.5

The estimated Quality Value (QV) of the final assembly is 59.3 with *k*-mer completeness of 100%, and the assembly has a BUSCO v5.3.2 completeness of 95.1% (single = 93.7%, duplicated = 1.4%), using the endopterygota_odb10 reference set ( *n* = 2,124).

Metadata for specimens, spectral estimates, sequencing runs, contaminants and pre-curation assembly statistics can be found at https://links.tol.sanger.ac.uk/species/446526.

## Genome annotation report

The *Athripsodes cinereus* genome assembly (GCA_947579605.1) was annotated using the Ensembl rapid annotation pipeline ( Table 1; https://rapid.ensembl.org/Athripsodes_cinereus_GCA_947579605.1/Info/Index). The resulting annotation includes 16,603 transcribed mRNAs 16,582 protein-coding genes.

## Methods

### Sample acquisition and nucleic acid extraction

*Athripsodes cinereus* specimens were collected from River Kym, Great Staughton, UK (latitude 52.27, longitude –0.35) on 2019-03-11, using a kick-net. The specimens were collected and identified by John Findlay (Environment Agency) and preserved on dry ice. The specimen with ID NHMUK014361235 (ToLID iiAthCine2) was used for DNA sequencing, and the specimen with ID NHMUK014361226 (ToLID iiAthCine4) was used for Hi-C scaffolding.

DNA was extracted at the Tree of Life laboratory, Wellcome Sanger Institute (WSI). The iiAthCine2 sample was weighed and dissected on dry ice with tissue set aside for Hi-C sequencing. Tissue from the whole organism was cryogenically disrupted to a fine powder using a Covaris cryoPREP Automated Dry Pulveriser, receiving multiple impacts. HMW DNA was sheared into an average fragment size of 12–20 kb in a Megaruptor 3 system with speed setting 30. Sheared DNA was purified by solid-phase reversible immobilisation using AMPure PB beads with a 1.8X ratio of beads to sample to remove the shorter fragments and concentrate the DNA sample. The concentration of the sheared and purified DNA was assessed using a Nanodrop spectrophotometer and Qubit Fluorometer and Qubit dsDNA High Sensitivity Assay kit. Fragment size distribution was evaluated by running the sample on the FemtoPulse system.

### Sequencing

Pacific Biosciences HiFi circular consensus DNA sequencing libraries were constructed according to the manufacturers’ instructions. DNA sequencing was performed by the Scientific Operations core at the WSI on a Pacific Biosciences SEQUEL II (HiFi) instrument. Hi-C data were also generated from tissue of iiAthCine4 using the Arimav2 kit and sequenced on the Illumina NovaSeq 6000 instrument.

### Genome assembly, curation and evaluation

Assembly was carried out with Hifiasm ( Cheng *et al.*, 2021) and haplotypic duplication was identified and removed with purge_dups ( Guan *et al.*, 2020). The assembly was then scaffolded with Hi-C data ( Rao *et al.*, 2014) using YaHS ( Zhou *et al.*, 2023). The assembly was checked for contamination and corrected using the gEVAL system ( Chow *et al.*, 2016) as described previously ( Howe *et al.*, 2021). Manual curation was performed using gEVAL, HiGlass ( Kerpedjiev *et al.*, 2018) and Pretext ( Harry, 2022). The mitochondrial genome was assembled using MitoHiFi ( Uliano-Silva *et al.*, 2022), which runs MitoFinder ( Allio *et al.*, 2020) or MITOS ( Bernt *et al.*, 2013) and uses these annotations to select the final mitochondrial contig and to ensure the general quality of the sequence.

A Hi-C map for the final assembly was produced using bwa-mem2 ( Vasimuddin *et al.*, 2019) in the Cooler file format ( Abdennur & Mirny, 2020). To assess the assembly metrics, the *k*-mer completeness and QV consensus quality values were calculated in Merqury ( Rhie *et al.*, 2020). This work was done using Nextflow ( Di Tommaso *et al.*, 2017) DSL2 pipelines “sanger-tol/readmapping” ( Surana *et al.*, 2023a) and “sanger-tol/genomenote” ( Surana *et al.*, 2023b). The genome was analysed within the BlobToolKit environment ( Challis *et al.*, 2020) and BUSCO scores ( Manni *et al.*, 2021; Simão *et al.*, 2015) were calculated.

Table 3 contains a list of relevant software tool versions and sources.

**Table 3.** Software tools: versions and sources.

Software tool	Version	Source
BlobToolKit	4.1.5	https://github.com/blobtoolkit/blobtoolkit
BUSCO	5.3.2	https://gitlab.com/ezlab/busco
gEVAL	N/A	https://geval.org.uk/
Hifiasm	0.16.1-r375	https://github.com/chhylp123/hifiasm
HiGlass	1.11.6	https://github.com/higlass/higlass
Merqury	MerquryFK	https://github.com/thegenemyers/MERQURY.FK
MitoHiFi	2	https://github.com/marcelauliano/MitoHiFi
PretextView	0.2	https://github.com/wtsi-hpag/PretextView
purge_dups	1.2.3	https://github.com/dfguan/purge_dups
sanger-tol/genomenote	v1.0	https://github.com/sanger-tol/genomenote
sanger-tol/readmapping	1.1.0	https://github.com/sanger-tol/readmapping/tree/1.1.0
YaHS	1.1a.2	https://github.com/c-zhou/yahs

### Genome annotation

The BRAKER2 pipeline ( Brůna *et al.*, 2021) was used in the default protein mode to generate annotation for the *Athripsodes cinereus* assembly (GCA_947579605.1). in Ensembl Rapid Release.

### Wellcome Sanger Institute – Legal and Governance

The materials that have contributed to this genome note have been supplied by a Darwin Tree of Life Partner. The submission of materials by a Darwin Tree of Life Partner is subject to the **‘Darwin Tree of Life Project Sampling Code of Practice’**, which can be found in full on the Darwin Tree of Life website here. By agreeing with and signing up to the Sampling Code of Practice, the Darwin Tree of Life Partner agrees they will meet the legal and ethical requirements and standards set out within this document in respect of all samples acquired for, and supplied to, the Darwin Tree of Life Project. 

Further, the Wellcome Sanger Institute employs a process whereby due diligence is carried out proportionate to the nature of the materials themselves, and the circumstances under which they have been/are to be collected and provided for use. The purpose of this is to address and mitigate any potential legal and/or ethical implications of receipt and use of the materials as part of the research project, and to ensure that in doing so we align with best practice wherever possible. The overarching areas of consideration are:

•   Ethical review of provenance and sourcing of the material

•   Legality of collection, transfer and use (national and international) 

Each transfer of samples is further undertaken according to a Research Collaboration Agreement or Material Transfer Agreement entered into by the Darwin Tree of Life Partner, Genome Research Limited (operating as the Wellcome Sanger Institute), and in some circumstances other Darwin Tree of Life collaborators.

## Data Availability

European Nucleotide Archive: *Athripsodes cinereus*. Accession number PRJEB57670; https://identifiers.org/ena.embl/PRJEB57670. ( Wellcome Sanger Institute, 2022) The genome sequence is released openly for reuse. The *Athripsodes cinereus* genome sequencing initiative is part of the Darwin Tree of Life (DToL) project. All raw sequence data and the assembly have been deposited in INSDC databases. Raw data and assembly accession identifiers are reported in Table 1.

## Author information

Members of the Natural History Museum Genome Acquisition Lab are listed here: https://doi.org/10.5281/zenodo.4790042.

Members of the Darwin Tree of Life Barcoding collective are listed here: https://doi.org/10.5281/zenodo.4893703.

Members of the Wellcome Sanger Institute Tree of Life programme are listed here: https://doi.org/10.5281/zenodo.4783585.

Members of Wellcome Sanger Institute Scientific Operations: DNA Pipelines collective are listed here: https://doi.org/10.5281/zenodo.4790455.

Members of the Tree of Life Core Informatics collective are listed here: https://doi.org/10.5281/zenodo.5013541.

Members of the Darwin Tree of Life Consortium are listed here: https://doi.org/10.5281/zenodo.4783558.
