# Supplementary material for: The genome sequence of the Brown Silverhorn, Athripsodes cinereus (Curtis, 1834)
Source: Wellcome Open Res. 2023 Sep 1;8:378. [Version 1] doi: 10.12688/wellcomeopenres.19765.1 (PMC11544197; doi:10.12688/wellcomeopenres.19765.1)
Supplement: Peer review history

# Reviewer report: Reviewer response for version 1

**DOI:** 10.21956/wellcomeopenres.21891.r106682

**Published:** 2024-11-07

**Authors:** Jerome H L Hui

**Affiliations:** 1 The Chinese University of Hong Kong, Hong Kong, Hong Kong

In this data note, Wallace and colleagues reported the genomic resource of caddisfly *Athripsodes cinereus *(Curtis, 1834). There are only limited amount of molecular data available for this species prior to this report (such as the mitochondrial COI sequences deposited to the NCBI database). Therefore, this new genome resource will be useful for further studies, ranging from revealing their biology, population structures, to understanding their evolution with other insects.

 This genome resource is excellent from the summary statistics, with high BUSCO number scores, high sequence continuity (scaffold N50), and majority of sequences contained on the 24 pseudochromosomes (plus sex chromosome and mitochondrion). All in all, this is a valuable contribution.

Are sufficient details of methods and materials provided to allow replication by others?

Yes

Is the rationale for creating the dataset(s) clearly described?

Yes

Are the datasets clearly presented in a useable and accessible format?

Yes

Are the protocols appropriate and is the work technically sound?

Yes

Reviewer Expertise:

Genomics, evolution, invertebrates

I confirm that I have read this submission and believe that I have an appropriate level of expertise to confirm that it is of an acceptable scientific standard.

# Reviewer report: Reviewer response for version 1

**DOI:** 10.21956/wellcomeopenres.21891.r92285

**Published:** 2024-08-12

**Authors:** Doga Cedden

**Affiliations:** 1 University of Göttingen, Göttingen, Germany

Wallace *et al*. report a genome assembly from *Athripsodes cinereus of *sufficient quality. The background information regarding the biology of the insect is provided. The methodology is sufficiently explained within the manuscript and is appropriate for the assembly. The authors conducted and reported sufficient quality analysis of their genome assembly. The deposited files are accessible.

1. The reported GC content for the mitochondrial genome is quite low (20.5%), which is also the case in some other insects. This can be highlighted in the manuscript.
2. As far as I understood, Fig.1 is a photograph by a third party with adequate attribution. However, it would be much preferred if the specimen used for the assembly was provided. This is just a note for subsequent assemblies and is not required for this data note.

Are sufficient details of methods and materials provided to allow replication by others?

Yes

Is the rationale for creating the dataset(s) clearly described?

Yes

Are the datasets clearly presented in a useable and accessible format?

Yes

Are the protocols appropriate and is the work technically sound?

Yes

Reviewer Expertise:

I work on coleopteran pests, mainly using RNA interference and RNA-seq.

I confirm that I have read this submission and believe that I have an appropriate level of expertise to confirm that it is of an acceptable scientific standard.
